# Gyroscope-Based Continuous Human Hand Gesture Recognition for Multi-Modal Wearable Input Device for Human Machine Interaction

**DOI:** 10.3390/s19112562

**Published:** 2019-06-05

**Authors:** Hobeom Han, Sang Won Yoon

**Affiliations:** Department of Automotive Engineering, Hanyang University, Seoul 04763, Korea; lucizn@icloud.com

**Keywords:** hand gesture, continuous gesture recognition, gyroscope, multi-modal input devices, unified wearable input devices

## Abstract

Human hand gestures are a widely accepted form of real-time input for devices providing a human-machine interface. However, hand gestures have limitations in terms of effectively conveying the complexity and diversity of human intentions. This study attempted to address these limitations by proposing a multi-modal input device, based on the observation that each application program requires different user intentions (and demanding functions) and the machine already acknowledges the running application. When the running application changes, the same gesture now offers a new function required in the new application, and thus, we can greatly reduce the number and complexity of required hand gestures. As a simple wearable sensor, we employ one miniature wireless three-axis gyroscope, the data of which are processed by correlation analysis with normalized covariance for continuous gesture recognition. Recognition accuracy is improved by considering both gesture patterns and signal strength and by incorporating a learning mode. In our system, six unit hand gestures successfully provide most functions offered by multiple input devices. The characteristics of our approach are automatically adjusted by acknowledging the application programs or learning user preferences. In three application programs, the approach shows good accuracy (90–96%), which is very promising in terms of designing a unified solution. Furthermore, the accuracy reaches 100% as the users become more familiar with the system.

## 1. Introduction

Recent innovations in electronics and wearable technologies facilitate interactive communication between human beings and machines, including computers. This human–machine interface (HMI) system will become more important for the Internet of Things (IoT) and ubiquitous computing [1]. Typically, communication starts when an object (i.e., a machine) receives and interprets a human’s (i.e., the user’s) intention. Thus, for the HMI, an input device that can capture the user’s intention is crucial.

Human gestures enable an ergonomic approach to input for the HMI. Human body language is an important communication tool that is intuitively used to convey, exchange, interpret, and understand people’s thoughts, intentions, or even emotions. Thus, body language not only supports or conveys emphasis in spoken language but also is a complete language in itself; it is natural to consider human gestures, such as hand gestures, for HMI input [2]. However, so that they can be widely accepted as a HMI input, recognition of human gestures still has several hurdles to overcome.

One critical challenge is that human hand gestures are significantly less diverse than the functions needed by the HMI. HMI functions are more diversified and complicated. This trend of diversification is observable in the smartphone example. Only a decade ago, several handheld electronic devices co-existed to cover diverse human needs, including cell phones, personal digital assistants (PDAs), mp3 players or CD players, digital cameras or digital camcorders, gaming devices, and calculators, whereas, now, almost all the functions of these devices converge into a single mobile device: A smartphone. In contrast, in a smartphone, all human intentions are expressed only by swiping or tapping fingers on the touch screen.

Hand gesture-based interaction is one common approach being considered as HMI inputs [3]. Hand gestures are recognized by two major methods: Vision image processing [4] or wearable electronics [5]. Vision sensors are popularly used, especially in specific applications, such as smart televisions [6] or multimedia applications [7]. Though, in the last decade, dramatic advances have been made in semiconductor sensors (e.g., micro electro mechanical systems sensors). These advances provide precise, small-sized, light-weighted, and low-priced sensor solutions that are “wearable” by human beings. 

Wearable sensors include electromyography (EMG), touch sensors, strain gauges, flex sensors, inertial sensors, and ultrasonic sensors [8]. Among wearable sensors, wearable inertial sensors may be the most widely employed for human-motion recognition [9,10]. In general, inertial sensors refer to sensor systems consisting of accelerometers and gyroscopes, and magnetometers.

It is common to co-use multiple wearable sensors by sensor-fusion algorithms. For example, a glove with multiple wearable sensors is reported to monitor hand gestures [11]. A 3D printer is used to manufacture the glove housing, which contains flex sensors (on fingers), pressure sensors (at fingertips), and an inertial sensor (on the back of one’s hand). 

In many sensor-fusion algorithms, inertial sensors are typically used to track hand motions, while other sensors (e.g., EMG sensors) detect additional hand information, such as finger snapping, hand gripping, or fingerspelling [12,13]. One prominent combination may be inertial and EMG sensors [12,13,14,15,16,17]. The hand position is determined by the inertial sensor and the EMG sensors provide supportive information to fully understand complicated finger or hand gestures. It is also possible to adopt strain gauges, tilt sensors, or even vision sensors, instead of the EMG sensors.

These recognition methods of complex gestures consequently increase the amount of sensor data. To handle the increased data, machine learning is drawing attention. Various machine learning techniques are introduced for wearable sensors. Data from a wristband device having EMG sensors are processed by either a linear discriminant analysis (LDA) classifier [13] or a support vector machine classifier [18]. In another study, signals generated from a MEMS accelerometer are digitized, coded, and analyzed using a feedforward neural network (FNN) [19]. 

Meanwhile, there have been approaches using only wearable inertial sensors. This inertial-sensor-only approach potentially increases portability and mobility with a reduced computation load, compared to the cases using multiple wearable sensors or heavy algorithms. A research team asked users to write words using a smartphone as a pen [20] and reconstruct the handwritings using a gyroscope and accelerometer embedded in the phone. The handwriting included English and Chinese characters and emoticons. Other studies utilized kinematics based on inertial sensor signals to monitor hands or arms [21,22,23]. Recognizing the motions of a head or feet are also reported [24,25] but they are not adapted in hand gesture recognition.

As input devices for a HMI, it cannot be doubted that wearable inertial sensors should be accurate and rapid. However, these dual goals are contradictory, because improved accuracy frequently increases the computation load, resulting in slow speed. In addition, user hand gestures should be simple and straightforward. Moreover, inertial-sensor-based gesture-recognition systems additionally have fundamental limitations. One limitation is the inertial sensor noise, which continues to be accumulated, resulting in bias or drift in the system output [26]. The second limitation is that signals from MEMS gyroscopes may be confused with accelerometer signals [27].

To resolve these problems, the signal processing of inertial sensor outputs has actively been investigated, from simple outputs (such as moving average filters) to the recently developed outputs (such as machine learning). Recent approaches include digitizing the sensor signals to generate codes and calculating statistical measures of the signals to represent their patterns. One system distinguished seven hand gestures using a three-axis MEMS accelerometer [28]. Accelerometer signals are digitized by labelling positive and negative signals and are restored by a Hopfield network.

These accelerometer-only approaches effectively capture linear gestures (e.g., up/down or left/right patterns), but are not easily applicable for detecting circular motions (e.g., clockwise rotation or hand waving). To recognize both linear and rotational gestures, methods that rely on both accelerometers and gyroscopes were proposed. A research applied the Markov chain algorithm to monitor the movement of the arms using accelerometer and gyroscope sensors worn on the forearms [29]. In another recent work paper, a real-time gesture recognition technique, named Continuous Hand Gestures (CHG), was reported [30]. The technique first defines six basic gestures, then finds their statistical measures, including means and standard deviations (STDs), and finally produces a database for the measures of each gesture. 

These accelerometer-gyroscope combinations exhibit an excellent accuracy, but still require solutions providing multiple functions with a limited number of hand gestures. To address these challenges, the objective of this study was to develop a unified multi-modal HMI input device conveying the user’s intention rapidly and precisely. A comparison with published works with other using sensors is summarized in Table 1.

Table 1 summarizes recent activities reporting the use of various wearable sensors as the HMI input, using the accelerometer, gyroscope, accelerometer-gyroscope fusion, ultrasonic, and fusion accelerometer-gyroscope with electromyography approaches. The accelerometer-only approach cannot detect rotational motions, and some computation loads should be allowed for the sensor fusion (depending on logics) or machine learning algorithms (during training). Thus, this paper selects a gyroscope-only system, expecting better rotation-sensing capability (than the accelerometer-only systems), reduced sensor cost and computation load (than the sensor fusion), and decreased computational load during model training (than the machine learning). Of course, these comparisons are only qualitative explanations and should acknowledge that the performance of each method can be improved by algorithm/system optimization. Trajectory tracking is also considered because it is the functionality equipped in laser pointers or computer mice. In addition, most recognition methods hold the gesture-signal data for a certain time, which is defined as the steady gesture state [23] in the table, to improve detection accuracy. However, for real-time HMI inputs, a continuous recognition is preferred. As all references in Table 1 report excellent recognition rates exceeding 90%, it is reasonable to target a recognition rate larger than 90%. Though, this work tries to enable multifunctional capabilities with less numbers of hand gestures, which are not seriously considered in all the references. This uniqueness is crucial for multi-modal HMI input devices, we think, and is expressed by the number of demonstrated applications in the table.

## 2. Design of the Wearable System

Our proposed system is configured to implement several important key features. First, our system relies on selected simple hand gestures (denoted as “unit gestures”), whose functions are redefined for different application programs. A machine (utilizing our wearable system) already acknowledges the currently running application. Therefore, the function executed by each gesture can differ by application, facilitating multifunction capabilities with less gesture complexity to realize a unified multi-modal input device for HMI.

The second feature is that the sensor used is simplified to use only one three-axis gyroscope which is, however, providing both gesture recognition and trajectory tracking functions. In addition, this approach miniaturizes the wearable devices and reduces required cost, compared to the accelerometer-gyroscope combination.

The third feature is continuous hand-gesture recognition in real time. To minimize the delay caused by computation load, we reduced the computational complexity by employing a simple algorithm that calculates the normalized covariance between the pattern signal of the user’s hand gesture and the reference signal pattern. Signal waveforms (generated during experiments) and their characteristics were stored in an in-built database with an appropriate window size.

The last feature is the system accuracy. Despite the fact that the complexity is reduced and multiple input devices converge into a single miniature device, sufficient accuracy should be guaranteed. To avoid errors caused by a hand tremor or unintentional hand gestures, we co-considered pattern similarity and signal magnitudes, and, through experiments, deduced the recognition threshold values that correctly identify the hand. In addition, a learning mode was included for user customization.

Our multi-modal input device is anticipated to be employed in various consumer electronics. Possible major applications include input devices to (1) computers, such as personal computers, laptops, or tablet PCs, (2) portable multimedia players like mp3 players or smartphones, (3) wireless remote controllers for presentation programs, home electronics, or video game consoles, and (4) a head mounted display (HMD) typically used in virtual reality modules. As an example, a user connects our multi-modal input device to a laptop and gives a presentation to audiences. After finishing the meeting, the user wants to read an article that he/she stopped reading for the meeting. While the user is waiting for a bus, he/she goes back to the previously viewed website and scrolls up to refresh news feeds. In the bus, the user watches a movie chip using a smartphone or HMD, and, after coming back home, the user wants to turn on an air conditioner and a robot vacuum cleaner. 

Even in this simple scenario, we require many input devices, such as a laser presentation remote, a computer mouse and keyboard, and several remote controllers. However, all of these can be replaced by a single multi-modal input device, which is the main target of our approach. To demonstrate the concept, we selected three example cases (giving a presentation, playing a video, and surfing a website) and conducted experiments using one input device. Details are described in Section 4.

**Figure 1:**
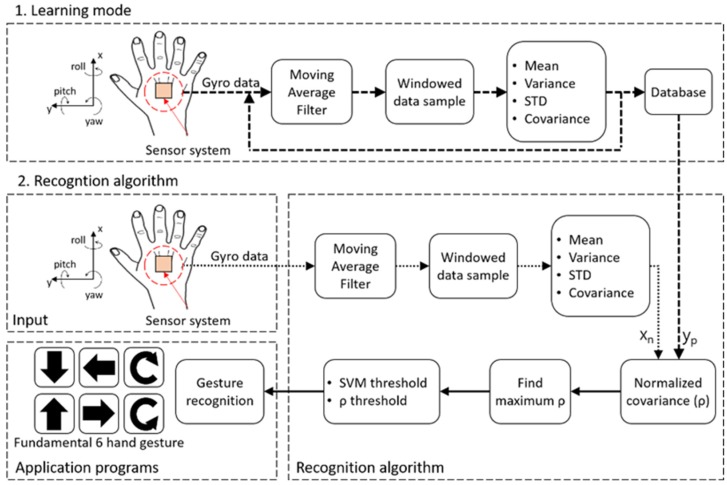
Flowchart of the system data.

An overview of the designed system with algorithms is shown in Figure 1. The gyroscope generates angular velocity data from hand gestures and feeds the data to the machine (e.g., a personal computer) interfaced with the three-axis gyroscope. The machine processes the raw sensor data using a custom-moving average filter to reduce sensor noise, produced either by the sensor limitations or unwanted gestures, such as hand tremors. In addition, initially, a learning mode is conducted so that the machine “learns” the preferences and habits of users. The reference signal pattern is updated and fitted according to the user’s gesture. 

We analyzed the characteristics of the data, including their average, standard deviation (STD), variance, and covariance values. The values were used to calculate a normalized covariance (*ρ*), which was the key determinant of gesture recognition in this study. To derive the analyzed values, the filtered data were windowed to select a set of data samples selected from the most recent data samples.

The normalized covariance was calculated for the unit gestures, respectively, and the gesture maximizing the *ρ* value was determined to be that which the user intended. The machine selected a gesture that maximized *ρ*. The signals of the six gestures were already learned by the machine in the learning mode, which is initiated when a user turns on the machine. Then, the sensor signal was compared with two thresholds (related to the signal vector magnitude (SVM) and *ρ*) to enhance recognition accuracy with a low computation load. The machine validated a gesture as an intended gesture through comparing the threshold values and the magnitude of the input signal. The details are described in the following sections.

## 3. Unit Hand Gesture Recognition Algorithm

### 3.1. Definition of Unit Hand Gestures

**Figure 2:**
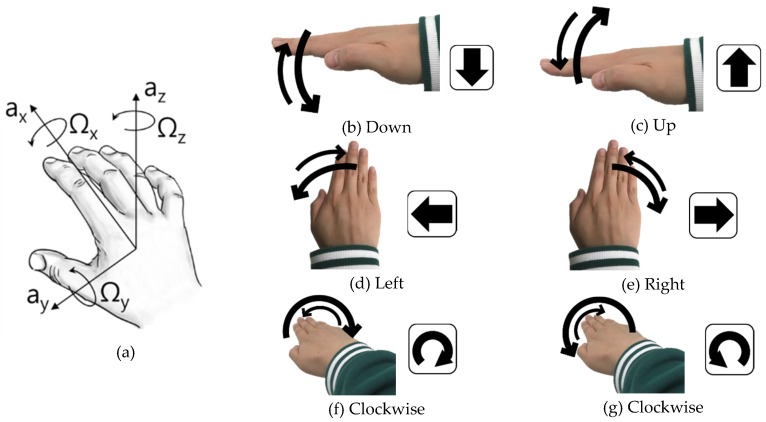
Six unit hand gestures selected based on user convenience. (**a**) The coordinate system and major directions of a wearable sensor mounted on a hand, (**b**) downward movement along the *y*-axis, (**c**) upward movement along the *y*-axis, (**d**) left directional movement along the *z*-axis, (**e**) right directional movement along the *z*-axis, (**f**) clockwise (CW) movement along the *x*-axis, and (**g**) counter clockwise (CCW) movement along the *x*-axis. Each thick arrow indicates the first motion direction and the thin arrow shows how the second (sequential) motion occurs to return to the neutral position.

As noted, the required functions of an input device for the HMI are diverse, but the number of available hand gestures (promising user convenience) are relatively limited. Figure 2 depicts six unit hand gestures that we selected based on the coordinate system. The coordinate system is a Cartesian coordinate system assuming that a wearable sensor is mounted on the back of the user’s hand or palm.

The six unit gestures are selected because there are x, y, and, *z*-axes and each axis has two rotational directions. In addition, these six hand gestures are those most commonly investigated in previous HMI research studies [28,30]. Note that the six unit gestures are used as building blocks (like English alphabets). Users have the choice to use the unit gestures by themselves or create their own gestures by sequentially combining them for new functions.

Figure 2a illustrates the three linear and three rotational directions required to capture the hand gestures. Human body motions are, in general, accomplished by rotating joints, and thus, sensor-wise, rotational detection is more reasonable and user-friendly than linear movement measurement [31]. Therefore, we decided to use a three-axis gyroscope, instead of both accelerometers and gyroscopes.

**Figure 3:**
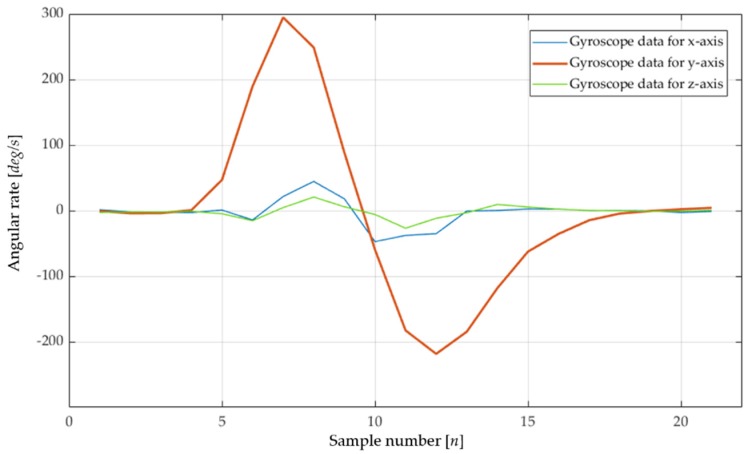
Experimented reference waveform patterns of the “Down” gesture having different magnitudes.

For the definition of reference waveform patterns, each unit gesture was repeated 100 times and data sets were averaged to define the reference waveform pattern for reference. Figure 3 depicts the reference waveforms of the “Down” gesture, which is one of the six unit gestures. The reason why the waveforms are bi-directional is that a user first moves his/her hand to the intended direction and then returns it to the neutral position. In this study, the maximum amplitude is not significantly meaningful, because the normalized covariance relies mainly on pattern similarity and not strongly on signal magnitudes.

Each unit gesture is observed to generate distinguishable reference patterns. The “Down” and “Up” gestures are dominated by the *y*-axis rotation, while the “Right” and “Left” gestures generate mainly z-axis rotation signals. The dominant signals of the clockwise (CW) and counter clockwise (CCW) are rotation around the *x*-axis. 

In addition to the difference in the dominant rotation axis, the signal polarity is also considered. For example, the CW and CCW gestures are similar, in that they are reciprocating rotation around the *x*-axis, but different in the polarity of the first rotation, which is positive in the CW gesture and negative in the CCW gesture. If a user consecutively rotates his/her wrist several times, this activity is considered a new combination gesture, different from the unit gestures.

### 3.2. Calculating Variables for Average, Standard Deviation, and Variance of the Filtered Signal

In this section, we state the assumption and define the variables used in this paper. Their notations are also summarized in Table 2. As depicted in Figure 1, our system starts with the acquisition of gyroscope data. Let *g*[*n*] *=* [*g_x_*[*n*], *g_y_*[*n*], *g_z_*[*n*]]*^T^* denote the raw gyroscope data at sample number *n* in each corresponding axis.

As explained, the sensor data may contain unwanted high-frequency data generated by unwanted hand movements. To avoid this problem, a moving average filter is used. The moving average filter is a filter that stores a certain number of data and corrects the output value by averaging them. Using the raw gyroscope data, *g*[*n*], the filter formula is:

(1) $$x_{m}=\frac{1}{m}\sum_{n=1}^{m}g[n]$$

As the *m* value increases, sensor noise is reduced, but a slower response rate is expected. In order to optimize the *m* value, this study adjusted the value to be a minimum of two to a maximum of five, depending on the operating speed of the running application programs.

It was reported that the energy of hand gestures is mostly located at signals having a frequency lower than 10 Hz [32]. Thus, in this study the sensor sampling frequency was set at 20 Hz. The filtered sensor data were recorded for 1 s (thus, the window size was 20 data samples. The window size was denoted by *N*) and their average, variance, and standard deviation (STD) were calculated, which are given in Equations (2)–(4). Let *x_m_*[*n*] *=* [*x_x_*[*n*], *x_y_*[*n*], *x_z_*[*n*]]*^T^* denote the moving average filtered gyroscope data at sample number *n*.

(2) $$\bar{x}=\frac{1}{N}\sum_{n=1}^{N}x_{m}[n]$$

(3) $$\sigma_{x}{}^{2}=\frac{1}{N}\sum_{n=1}^{N}{\left(x_{m}[n]-\bar{x}\right)}^{2}$$

(4) $$\sigma_{x}=\sqrt{\frac{1}{N}\sum_{n=1}^{N}{\left(x_{m}[n]-\bar{x}\right)}^{2}}$$

The standard deviation is a measure of the distribution of the signal from the average of the signal. The covariance value is a coefficient, indicating the variance and directionality of the combined signal distribution of the distribution of two signals.

When a user first uses the sensor system, a special algorithm named “a learning mode” operates, as shown in Figure 1. In the learning mode, a user performs unit gestures, and the waveform patterns of each average-filtered sensor signal are recorded as reference signals (denoted *y_p_*, where *p* = 1,2,3,4,5,6. Each integer corresponds to each unit gesture). Thus, using the reference signals, the sensor system learns the user’s habits, tendency, or preference.

The learned reference (i.e., *y_p_*) and the sensor signal updated at a certain time (i.e., *x_m_*) were compared to determine their correlation. If the sensor signal matches with a reference signal of a specific hand gesture, we conclude that a user performed the specific gesture. For the comparison, we employed a normalized covariance given by

(5) $$\rho_{xy}=\frac{E[x_{m}-\bar{x},y_{p}-\bar{y}]}{\sqrt{E{\left[x_{m}-\bar{x}\right]}^{2}}\times \sqrt{E{\left[y_{p}-\bar{y}\right]}^{2}}}=\frac{\sigma_{xy}}{\sigma_{x}\times \sigma_{y}}$$

The normalized covariance was also called a correlation coefficient and provided a measure of similarity between the two signals. *σ_xy_* is the covariance about *x_m_* and *y_p_*. The calculated normalized covariance had a value from −1 to 1. A value of 1 meant that the two signals (*x_m_* and *y_p_*) had an identical waveform pattern, although their amplitudes or phases may have differed. If the normalized covariance is zero, there is no linear relationship between the two signals, which are independent of each other. However, note that the normalized covariance determines only the waveform pattern of hand gestures, but cannot judge the magnitude of gesture signals. Thus, there was a chance that a small signal, which, for example, could occur as a result of unintended gestures, such as hand tumors, could be detected.

### 3.3. Definition of the Minimum Threshold for the Normalized Covariance and SVM

To resolve this problem, we also considered the absolute of the recognized hand-gesture signal. For a successful recognition, the *ρ* value should exceed a pre-determined threshold value (*ρ_th_*) and the average value of a signal vector magnitude (*M*, given in Equation (6)) of the signal should be larger than a threshold SVM value given by (*M_th_*). Magnitude of the normalized covariance is:

(6) $$M=\frac{1}{N}\sum_{n=1}^{N}\sqrt{x_{m,x}{\left[n\right]}^{2}+x_{m,y}{\left[n\right]}^{2}+x_{m,z}{\left[n\right]}^{2}}$$

Table 3 shows the results of the process for defining the minimum *ρ_xy_* value of each gesture. In the table, a user conducted a “Right” hand gesture and generated a sensor signal (*x_m_*), which is sequentially compared with the six reference waveform patterns (*y_p_*, *p* = 1,2,3,4,5,6), and their normalized covariance (*ρ_xy_*) is individually calculated. If the calculated *ρ*_xy_ is larger than the pre-defined threshold value (from 0.2 to 0.9), the *x_m_* signal is recognized to be its corresponding hand gesture.

This process was repeated 100 times at each threshold value and the probability of recognitions was calculated. Note that in a small (minimum) threshold value, one user-generated signal may have more than one similar pattern and thus be mistakenly recognized as two or more gestures. When the threshold value is 0.4 or less, the probability of recognizing the gesture in the opposite direction is rather high. When the range of the threshold value is larger than at least 0.55, all cases are correctly recognized as the “Right” gesture. Thus, in this user generated data in Table 3, the *ρ_th_* is set as 0.55. These observations suggest that we need to set the minimum threshold of the normalized covariance. 

Although the normalized covariance readily recognizes pattern similarity and difference, more information is still required for an accurate decision. For example, the“Down” gestures in Figure 3 (having different amplitudes) are all recognized as the “Down” gesture (due to high *ρ_xy_*), but should be differentiated because the small gesture in the figure may be generated by unideal cases, such as hand tremors or sensor noise. To avoid such errors, we needed to incorporate another decision factor, specifying signal amplitude.

**Figure 4:**
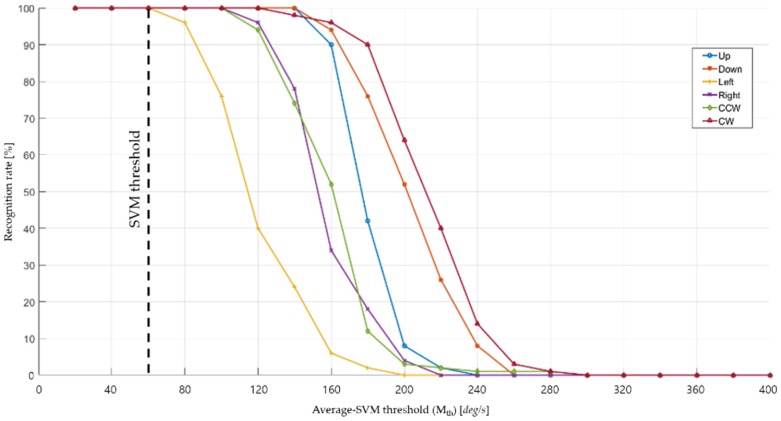
Recognition rates of each hand gesture by the function of threshold average SVM values.

The signal-amplitude factor employed herein is an averaged SVM, the derivation of which is given in Equation (6). SVM is popularly used in many applications, including machine learning or gait sensing [33,34]. Figure 4 illustrates the results of a process to determine the averaged-SVM threshold (*M_th_*) and depicts recognition rates of the six hand gestures with different *M_th_* values. Note that the *M_th_* values are basically angular velocities (unit of degree per second) because the sensor output source is a three-axis gyroscope. We first set the *M_th_* value at 20°/s, conducted the six hand gestures, and computed and compared their recognition rates. Then, we increased *M_th_* from 20°/s to 400°/s by steps of 20°/s and plotted the figure. It is noteworthy that we should select the smallest *M_th_* value, which is 60°/s in this figure, for 100% recognition. Thus, gestures having an averaged SVM smaller than *M_th_* are considered not to have occurred.

### 3.4. Validation of the Unit Gesture Recognition Algorithm

To validate the usefulness of the developed algorithm, the reference waveform patterns are experimentally analyzed. Another normalize covariance *ρ_r1r_*_2_ is defined as:

(7) $$\rho_{r_{1}r_{2}}=\frac{E[r_{1}-\bar{r_{1}},r_{2}-\bar{r_{2}}]}{\sqrt{E{\left[r_{1}-\bar{r_{1}}\right]}^{2}}\times \sqrt{E{\left[r_{2}-\bar{r_{2}}\right]}^{2}}}=\frac{\sigma_{r_{1}r_{2}}}{\sigma_{r_{1}}\times \sigma_{r_{2}}}$$

where, *r*_1_ and *r*_2_ are one of the waveform patterns of the unit gestures in Figure 2. Note that *ρ_xy_* in Equation (5) is the normalized covariance of the reference (*y_p_*) and user generated sensor output (*x_m_*), whereas the *ρ_r1r_*_2_ is the normalized covariance between two reference patterns (*r*_1_ and *r*_2_, where *r*_1_ = *y_p_*_1_ and *r*_2_ = *y_p_*_2_). 

Table 4 shows the recognition counts and rates of the unit gestures. After completing user customization (by the learning mode and threshold adjustments), each gesture set was conducted for 400 times by four users. In all cases, high recognition rates (96–97.65%) were achieved. This result implied that all hand gestures were independent, and thus, their waveform patterns were exclusively recognized and the developed unit-gesture-recognition algorithm was reliable.

## 4. Experimental Demonstration of Multimodal Capability

Previously, we described several key techniques, including normalized covariance for pattern recognition, two thresholds (*ρ_th_* and *M_th_*) for enhanced accuracy, and the learning mode for user-customized interaction. These techniques were used together to realize a multi-modal input device for the HMI, facilitating simple, real-time, accurate, user-friendly, and multi-functional features. These advantages were demonstrated by follow-up experiments. 

**Figure 5:**
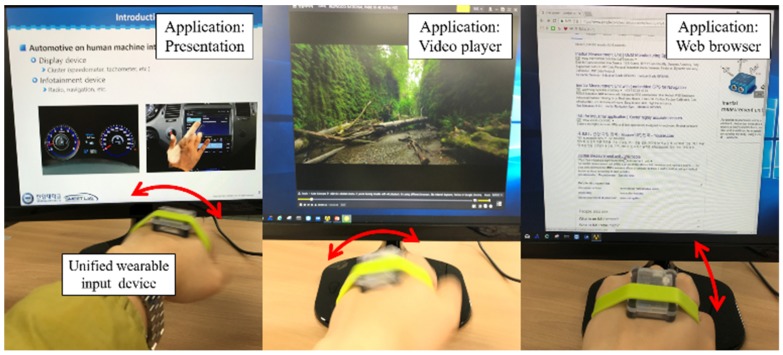
The unified multi-modal input device used for three application programs.

Our experimental setup is depicted in Figure 5. The sensor system was an inertial sensor system made of a micro controller unit, 2.4 GHz band chipsets, and a nine-axis inertial sensor. The inertial sensor included an accelerometer, a gyroscope, and a magnetometer, but this study only relied on the three-axis gyroscope. The gyroscope sampling rate was 20 Hz. The sensor system was assembled in a plastic box and communicated with the receiver.

### 4.1. Verification Using Three Different Application Programs

To verify the proposed concept, the developed input device was employed in three application programs, which were in general controlled by different input devices. The programs were the presentation program (the typical input device of which is a laser presentation pointer), a media player for playing video/movie files (the typical input device of which is a remote controller), and a web browser for surfing (the typical input device of which is a computer mouse).

Each experiment followed a predefined sequence. First, we ran the target application program and determined its core functions. Then, the functions were matched with the six hand gestures in Figure 2 and, if needed, simple combinations of the six gestures (e.g., two times “Right” gestures) were also used. When the initial setup had been completed, the first participant in the experiment operated the learning mode and then conducted a scenario comprising successive hand gestures executing all core functions. After the participant finished the scenario, the next participant followed the learning mode, which re-adjusted the input device according to his/her preferences, and conducted the scenario again. Five participants took part in this experiment.

This section may be divided by subheadings. It should provide a concise and precise description of the experimental results, their interpretation, as well as the experimental conclusions that can be drawn.

### 4.2. Application Program #1: Presentation

When giving a presentation, a user usually brings a presentation laser pointer and needs three major features. The first feature advances the presentation to the next slide or returns it to the previous slide. Sometimes, the user wants to return to the first slide or skip to the last slide to save slide-changing time. The second feature selects and runs objects embedded in a slide. The objects include movie clips, audio files, animations, etc. The final feature is used to draw the audience’s attention; it turns on a laser used for pointing at intended locations on a slide. This feature is called the focus mode and is exemplified by the laser-pointer option used in the slide-show mode. Based on this analysis, we selected seven key functions and matched them with the six hand gestures. The function-gesture matching results are listed in Table 5.

**Figure 6:**
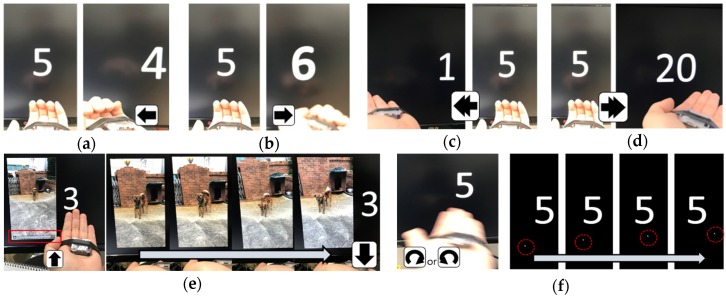
Sequence of experiments, (**a**) going to the previous slide, (**b**) going to the next slide, (**c**) jumping to the first slide, (**d**) jumping to the last slide, numbered 20, (**e**) selecting and executing a video clip, (**f**) activating the focus mode and moving the mouse cursor as a pointer.

These functions were experimented with, as shown in Figure 6. The arrow signs in the figure indicate the executed hand gestures. The number shown on the screen is the slide number. First, a participant was asked to conduct a “Left” gesture and the slide returned to the previous slide and the slide number changed from 5 to 4 (Figure 6a). When the participant was asked to execute a “Right” gesture, the slide number increased from slide 5 to 6 (Figure 6b). For faster transition, the participant rapidly conducted two “Left” or two “Right” gestures. This one-time “Double-Left” or “Double-Right” action resulted in the presentation going to the first slide (Figure 6c) or jumping to the final (20th) slide (Figure 6d). Then, the participant was asked to play a video clip embedded in slid 3. After conducting two slow “Left” gestures to go to slid 3, he/she performed an “Up” gesture to select the chip and sequentially made a “Down” gesture to play it (Figure 6e). While the video played, the participant rested his/her hand. Finally, the participant was asked to emphasize some contents in slide number 5. He/she conducted two slow “Right” gestures to go to the fifth slide and either a “CW” or a “CCW” gesture to activate the focus mode. As illustrated in Figure 6f, he/she then freely moved the mouse cursor (the white cursor movement is highlighted by the red circles). When the participant no longer needed the focus mode, he/she performed a “CW” or “CCW” gesture one more time and deactivated the mode.

Table 5 summarizes the success/error rates after five participants completed the sequence in Figure 6, 50 times. The error sources were individually analyzed by non-recognition (when a gesture is not recognized) and incorrect recognition (when it is recognized as a different gesture). The table shows that, regardless of the user, high success rates are demonstrated in the range of 92% to 96%. Thus, our single-device concept successfully incorporates all the needed functions of a presentation laser pointer and is suitable for use with a presentation.

### 4.3. Application Program #2: Playing Video/Music Files Using a Multimedia Player

Multimedia contents are mostly controlled by a remote controller. Multimedia controllers require four major features. The first feature is playing and pausing the currently playing file. The second feature is time shifting, such as fast-forwarding and rewinding, while the third is changing files in a playlist, such as playing the previous or next file. The final feature is volume control. Table 6 shows the function-gesture matching results of a multimedia player.

**Figure 7:**
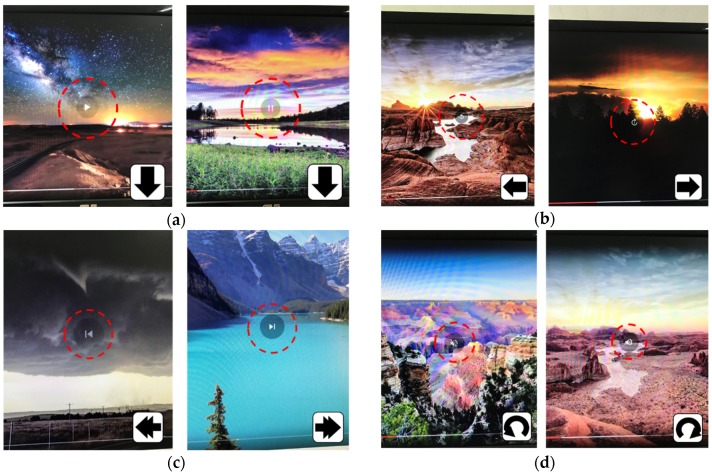
Sequence of experiments using gestures as input to a multimedia video player. (**a**) Playing and pausing functions, (**b**) time-shifting, either rewinding (left) or fast-forwarding (right), (**c**) playing previous (left) or next file in a playlist, (**d**) volume down (left) or up (right).

Figure 7 depicts an experimental sequence of playing a horizon-landscape video file. The red-circled symbol in each figure is generated by the used multimedia software and confirms which function is currently executed. A participant conducts a “Down” gesture to play the video and a second “Down” to pause it (Figure 7a). Then, he/she makes a “Left” gesture to rewind the video clip, the time of which goes back to dawn, and then performs a “Right” gesture to fast-forward the video so that its time rapidly goes to sunset (Figure 7b). Figure 7c illustrates the results of the “Double-Left” and “Double-Right” gestures: The video changes to the previous clip and the next video clips, respectively. Finally, in Figure 7d, the participant turns his/her hand counterclockwise and the video-sound volume decreases and eventually is muted. Then, he/she rotates the hand clockwise and increases the sound volume.

Table 6 reveals that the success rates span from 90% to 96%. The double-gestures (“Double-Right” or “Double-Left”) show the lowest success rate, because their non-recognition rate is relatively high. However, regardless of the participant or function, all gestures show superior success rates higher than or equal to 90%, which satisfies all the needed functions of multimedia players.

### 4.4. Application Program #3: Web-Surfing Using Web-Browser

As compared with the two application programs discussed in the previous sections, surfing web browsers requires different input characteristics, which are usually provided by a computer mouse and, if needed, the support of a computer keyboard. A conventional computer mouse provides two major features. One feature is selection functions provided by left or right clicks. The other is positioning the mouse cursor by moving the mouse. Keyboard functions may include going to the previous page (backspace key) and to the next page (alt-right-arrow keys) or refreshing the current page (F5 key). Table 7 is the gesture-function matching results of web-surfing.

**Figure 8:**
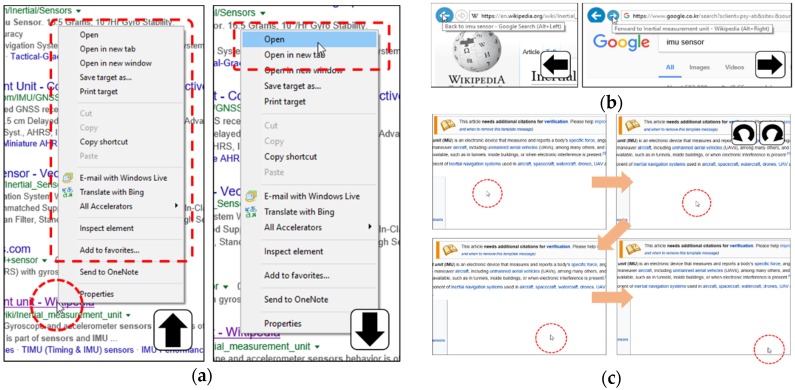
Practical test with web browser (**a**) left and right click, (**b**) back and forward, and (**c**) converting to the position mode.

Figure 8 illustrates an experiment sequence using the web browser. A participant first uses a default mode and locates the cursor on an intended website hyperlink. If the cursor does not move for a certain time (set as 0.7 s in this application), it freezes at the cursor-pointing location and allows the user to select certain activities. Then, the user conducts an “Up” gesture in Figure 8a. Now, the participant can move the cursor, meaning that the function changes to the cursor-positioning mode, and place it on the pop-up menu. He/she selects the function by making a “Down” gesture. In this step, he/she opens a page in the same tab. When the (selected) page is displayed, the participant makes a “Left” gesture to return to the previous page (here, the search page) and then performs a “Right” gesture to move to the forward page, as depicted in Figure 8b. When a user no longer wants to use left/right clicking or forward/backward functions, he/she can rotate his/her hand in either the clockwise or counterclockwise direction to return to the cursor-positioning mode (Figure 8c).

The experimental results are summarized in Table 7, showing that a high success rate is achieved in all functions, from 93% to 99%. Therefore, it is demonstrated that our concept can cover not only all the functions of a computer mouse but also some functions that require a computer keyboard.

### 4.5. Summary of the Verification Experiments

As noted, threshold values of normalized covariance (*ρ_th_*) and averaged SVM (*M_th_*) were adjusted by applications. For the presentation, *ρ_th_* is 0.8 and *M_th_* is 50. Both of them were relatively large, because a user typically used large gestures (large *M_th_*) during presentation and willingly accepted a lack of recognition but strongly wanted to avoid any wrong actions (large *ρ_th_*). Whereas, when a person listens to music or watches a video, hand gestures are typically large but a user is less concerned with incorrect recognition, which is easily corrected by quickly executing the right gesture one more time. Thus, the *M_th_* was maintained at 50, while the *ρ_th_* was decreased to 0.5. When a user surfs the web, the user’s hand movements show a wide speed range. Thus, the *M_th_* was decreased to 30. The normalized covariance threshold was increased to 0.7.

**Figure 9:**
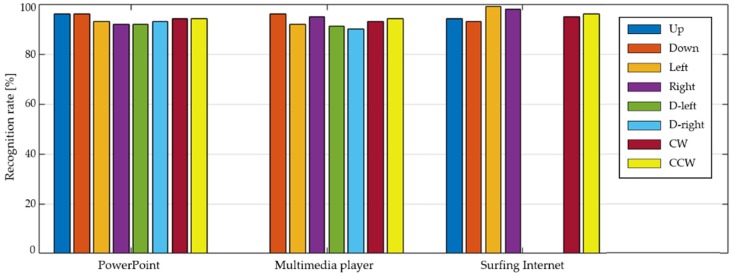
The recognition rates each function in three application programs.

Figure 9 summarizes the experimental results on gesture recognition rate in each program. All the recognition rates were higher than 90% and generally 92–96%. Moreover, the success rate increased as a user became more familiar with the input device. One accidental observation was that a user became more familiar with using our wearable input device and began to adapt himself/herself. This observation offered a hint for achieving a 100% success as the number of repetitions increased.

The computation load of this system was represented by a recognition delay time, which is experimentally evaluated herein. The delay time was defined as the time elapsed until a computer executed a specific function (matched to a specific gesture) since a user completed the corresponding hand gesture. The elapsed time was repeatedly measured for 100 times using a stopwatch. The measured delay-time values were 0.21 ± 0.05 s, and dominantly observed from 0.22 to 0.23 s. These values were not significantly long and were within the time scale of the cognitive band (0.1 to 10 s), which is the time required for a computer mediated HMI system [35]. Thus, we consider our system to be able to operate in real time.

## 5. Conclusions

This paper proposes a method providing a wearable electronics system providing a unified multi-modal input device for HMI systems. Six unit gestures are employed and resynchronized for three different application programs. The resynchronization is feasible because a machine in an HMI system already recognizes which program is currently running, and the required functions differ according to application programs. The resynchronization-by-program approach reduces the number of required functions to a great extent and (sequentially) the diversity in HMI input devices, realizing a unified (multi-modal) input device for HMI systems with less complex hand gestures. 

For fast and reliable recognition, two determinants are used: Normalized covariance and averaged SVM. The normalized covariance determines the gesture pattern similarity, and the SVM distinguishes errors caused by small hand gestures. In addition, the machine initially learns user preferences and habits by means of a learning mode. Thus, a highly successful gesture-recognition algorithm is achieved. 

The developed algorithm was applied to three application programs: Presentations, a multimedia player (for playing video/music files), and a web browser. The three programs are usually controlled by a laser pointer, remote controller, and computer mouse, respectively. Our single wearable sensor exhibits high success rates for the different functions of the three programs. Therefore, the developed sensor has high potential as a multi-modal wearable input device for HMI systems.

## Figures and Tables

**Table 1:** Summary of related works regarding wearable sensor-based gesture recognition.

Reference	Used Sensor(s)	Trajectory Tracking ^1^	Gesture State	Number of Gestures	Recognition Methods (Specific Methods, if any) ^2^	Demonstrated Applications
Xu et al., 2012 [28]	Accelerometer	No	Steady	7	HMM, Hopfield network	1
Arsenault et al., 2015 [29]	Accelerometer, gyroscope	No	Steady	6	HMM, Markov chain	1
Xie et al., 2016 [19]	Accelerometer	No	Steady	8	Machine learning (FNN, SM)	1
Zhou et al., 2016 [25]	Accelerometer, gyroscope	No	Steady	5	Machine learning (DT, KNN, SVM)	1
Gupta et al., 2016 [30]	Accelerometer, gyroscope	No	Continuous	6	DTW	1
Wu et al., 2016 [22]	Gyroscope	No	Steady	12	Movement likelihood matrix updating	1
Yang et al., 2018 [8]	Ultrasonic	No	Continuous	11	Machine learning (LDA, support vector machine)	1
Jiang et al., 2018 [13]	Accelerometer, gyroscope, electromyography	No	Continuous	8	Machine learning (LDA)	1
This study	Gyroscope	Yes	Continuous	12 ^3^, (3 applications programs)	Normalized covariance & threshold adjustment	3

^1^ Our study simultaneously considers both trajectory tracking (used to position a presentation pointer on a computer screen, etc.) and gesture-pattern recognition (used to return to the previous presentation slide, etc.). In this study, presentation and web-surfing applications require this characteristic. ^2^ The used classifiers in machine learning. ^3^ The number of gestures includes the six-unit gestures and six two-time repeating gestures (e.g., double-left gesture). HMM (Hidden Markov model), FNN (Feedforward Neural Network), SM (Similarity matching), DT (Decision tree), KNN, (k Nearest neighbors), LDA (linear discriminant analysis).

**Table 2:** Notation in this paper.

Symbol	Description
*x*, *y*, *z*	Three major axes in a global coordinate
*n*, *m*, *p*	Integer variables
*g*[*n*]	Raw data of gyroscope
*x_m_*	Moving average data of *g*[*n*]
*y_p_*	Reference stored in the database after a learning mode
*N*	Sample number of window sized
$\bar{x}$	Average of *x_m_*
*σ_x_*	Variance of *x_m_*
*σ_x_* ^2^	Standard deviation of *x_m_*
*σ_xy_*	Covariance of *x_m_* and *y_p_*
*ρ_xy_*	Normalized covariance of *x_m_* and *y_p_*
*E*[*x*]	Expectation of *x_m_*
*M*	Average of SVM

**Table 3:** Recognition probability of the “Right” gesture depends on the threshold value.

*ρ_th_*	Down	Up	Left	Right	CW	CCW
0.20	19.2	1	46.1	33.7	0	0
0.25	4.5	0	57.6	37.9	0	0
0.30	0	0	62.4	37.6	0	0
0.35	0	0	63.1	36.9	0	0
0.40	0	0	64.3	35.7	0	0
0.45	0	0	49.5	50.5	0	0
0.50	0	0	8.3	91.7	0	0
0.55	0	0	0	100	0	0
0.60	0	0	0	100	0	0
0.65	0	0	0	100	0	0
0.70	0	0	0	100	0	0
0.75	0	0	0	100	0	0
0.80	0	0	0	100	0	0
0.85	0	0	0	100	0	0
0.90	0	0	0	100	0	0

The number is the percentage of counted numbers of each gesture.

**Table 4:** Recognition counts and rates of the gestures conducted by four users (trial number is 400).

	Down	Up	Left	Right	CW	CCW	Recognition Rate
Down	389	4	0	0	0	0	97.25%
Up	0	386	0	0	0	0	96.50%
Left	0	0	388	0	0	0	97.00%
Right	0	0	1	390	0	0	97.50%
CW	0	0	0	0	384	10	96.00%
CCW	0	0	0	0	0	391	97.75%

**Table 5:** Result of the presentation software (participant = 5; trials = 50).

Gestures	Functions	Success Rate	Error Rate	
			Non-Recognition	Incorrect Recognition
Up	Select an embedded object	96%	4%	0%
Down	Execute the selected object	96%	4%	0%
Left	Return to the previous slide	93%	5%	2%
Right	Advance to the next slide	92%	6%	2%
D-left	Jump to the first slide	92%	4%	4%
D-Right	Jump to the last slide	93%	2%	5%
CW	Switch to focus mode	94%	-	6%
CCW	Switch to focus mode	94%	-	6%

**Table 6:** Result of the multimedia playing software (participant = 5; trials = 50).

Gestures	Functions	Success Rate	Error Rate	
			Non-Recognition	Incorrect Recognition
Down	Play/Pause	96%	2%	2%
Left	Rewind by 10 s	92%	6%	2%
Right	Fast-forward by 10 s	95%	4%	1%
D-Left	Previous file in playlist	91%	6%	3%
D-Right	Next file in play list	90%	6%	4%
CW	Volume down	93%	2%	5%
CCW	Volume up	94%	1%	5%

**Table 7:** Result of the web surfing experiment (participant = 5; trials = 50).

Gestures	Functions	Success Rate	Error Rate	
			Non-Recognition	Incorrect Recognition
Up	Right click	94%	2%	4%
Down	Left click	93%	2%	5%
Left	Back to the previous page	99%	1%	0%
Right	Forward to the next page	98%	0%	2%
CW	Switch to cursor positioning	95%	3%	2%
CCW	Switch to cursor positioning	96%	2%	2%
